# Detection of synovitis in early knee osteoarthritis by MRI and serum biomarkers in Japanese general population

**DOI:** 10.1038/s41598-020-69328-w

**Published:** 2020-07-23

**Authors:** Kyota Ishibashi, Eiji Sasaki, Seiya Ota, Daisuke Chiba, Yuji Yamamoto, Eiichi Tsuda, Sugimura Yoshikuni, Kazushige Ihara, Yasuyuki Ishibashi

**Affiliations:** 1 Department of Orthopaedic Surgery, Hirosaki University Graduate School of Medicine, 5 Zaifu-cho, Hirosaki, Aomori 036-8562 Japan; 2 Department of Rehabilitation Medicine, Hirosaki University Graduate School of Medicine, Hirosaki, Japan; 3 Department of Social Medicine, Hirosaki University Graduate School of Medicine, Hirosaki, Japan

**Keywords:** Osteoarthritis, Diagnostic markers, Pathology

## Abstract

To investigate synovitis’ influence on early knee osteoarthritis (EKOA) by serum biomarkers and magnetic resonance imaging (MRI) findings in Japanese women. We enrolled 255 women aged 30–70 without radiographic abnormalities (Kellgren–Lawrence grade ≥ 2). Knee injury, OA outcome scores (KOOS), clinical examinations, and standing radiograph were used for classification criteria of EKOA. Participants were classified into normal knee group and EKOA group. All participants underwent MRIs of their right knee. The amount of effusion-synovitis volume was quantified. We compared serum matrix metalloproteinases-3 levels (MMP-3), high-sensitivity C-reactive protein, interleukin-6, and adiponectin between the groups. The relationship between synovitis and EOKA was investigated using multiple linear regression. Fifty-four participants (21%) were classified as EKOA. Serum MMP-3 concentration and effusion-synovitis volume were higher in patients with EKOA (*p* = 0.025 and *p* = 0.001, respectively). Effusion-synovitis volume negatively correlated with all KOOS subscales and positively correlated with serum MMP-3 concentration. Serum MMP-3 concentration was associated with effusion-synovitis volume β = 0.60, *p* < 0.001). There was mildly active but definitive synovitis in EKOA. This was an observational study so that no conclusions can be drawn regarding cause-effect for synovitis and symptoms. Future studies should conduct follow-up of participants with synovitis to assess the progression of knee OA.

## Introduction

Knee osteoarthritis (OA) is a major joint disease that causes chronic pain, stiffness, and disability, especially in the aging population^1,2^. It requires high-cost treatment, such as arthroplasty, decreases productivity, and creates absence from work; therefore, knee OA imposes a substantial and growing burden on society^3^. Although early intervention or preventive approaches are needed, the timing and identifying methodology are not established, as conventional standard diagnosis and assessment are conducted mainly based on the Kellgren–Lawrence (KL) grading on radiographs^4^, thus making it difficult to detect early and minute changes.

Recently, new criteria for detecting early knee OA were proposed by the first international EKOA workshop (EKOA) without radiographic abnormalities^5^; they allow identification of people with moderate knee symptoms who have same risk factors as those with definitive knee OA. EKOA prevalence was 9.5% in men and 15.0% in women, and the highest prevalence was noted in middle-aged females^6^. Nevertheless, EKOA’s etiology has not been well studied.

Several biomarkers are frequently used to assess disease activity precisely and quantitatively. There are reports regarding the availability of serum biomarkers to evaluate synovitis at an early phase and to predict OA progression. Interleukin-6 (IL-6) and keratan sulfate levels increase in the early phase of knee OA accompanied by knee pain^7,8^. Magnetic resonance imaging (MRI) is a useful imaging biomarker for assessing early phase of knee OA. The Framingham osteoarthritis study suggested that the prevalence of any abnormality was seen in 89% of participants without radiographic abnormalities^9^. These findings suggest that MRI can detect features suggestive of knee osteoarthritis that cannot be visualized on conventional radiographs; nevertheless, the correlation between MRI findings and EKOA detected using the new criteria is unclear.

Synovitis is an important factor for determining the incidence and progression of knee OA^10^. Inflamed synovium secretes proteases such as MMP-3 and disintegrins and metalloproteinases with thrombospondin motifs (ADAMTSs), as well as cytokines such interleukin 1β and tumor necrosis factor α, all of which damage the cartilage matrix^11^. Evidence of synovitis using serum biomarkers^8,12,13^, MRI^14–16^, and arthroscopic findings^9^ are related to the rapid progression of knee OA; nevertheless, these examinations are contraindicated in clinical practice and large epidemiological studies because they are expensive and time-consuming. Some studies investigate the importance of biomarkers in those with early stage of knee OA in large sample cohort studies^7,10^. Elucidating the prevalence of synovitis in patients with symptomatic knees without radiographic abnormalities and diagnostic serum biomarkers may lead to a better understanding of the pathology and the potential for therapeutic intervention.

We focused on middle-aged women in our large sample cohort of the general Japanese population to identify those at high-risk for developing knee OA. We investigated relationships among serum biomarkers of synovitis and MRI findings. Furthermore, we compared serum biomarker concentrations and synovitis activity as evaluated by MRI between normal knees and EKOA in middle-aged women. We hypothesized that the presence of synovitis would be demonstrated in those with EKOA, and that the severity of symptoms would correlate with synovitis biomarkers, even in individuals without radiographic abnormalities.

## Results

The number of participants with normal knee were 201(79%), and with EKOA were 54 (21%); the number of participants diagnosed with EKOA was 2 in the 30–39-year-old group, 14 in the 40–49-year-old group, 19 in the 50–59-year-old group and 12 in the 60–70-year-old group; the mean age was 54.5 ± 9.3 years, and the mean BMI was 22.1 ± 3.1 kg/m^2^; finally, age and BMI showed no significant difference between the normal and EKOA groups (*p* = 0.341 and *p* = 0.299, respectively) (Table 1).

**Table 1:** Clinical characteristics of participants.

	Total sample (n = 255)	Normal (n = 201)	EKOA (n = 54)	*p* value
Age, years	54.5 ± 9.3	54.3 ± 9.4	55.6 ± 8.9	0.481
BMI, kg/m^2^	22.1 ± 3.1	21.6 ± 3.2	23.0 ± 3.5	0.524
**KOOS**				
Symptom	91.2 ± 11.6	95.1 ± 6.2	76.9 ± 15.5	< 0.001
Pain	92.1 ± 13.0	97.0 ± 6.3	73.8 ± 15.1	< 0.001
ADL short ver	93.8 ± 12.6	97.9 ± 5.7	86.1 ± 14.9	< 0.001
QOL	83.4 ± 19.9	90.5 ± 13.3	56.8 ± 18.0	< 0.001
KL grade 0, n (%)	133 (52.2)	110 (54.7)	23 (42.6)	0.176

The values represent demographic data of all participants, participants with early knee osteoarthritis (EKOA), and those without EKOA. Data are presented as mean ± SD for age, BMI, and KOOS. The data of Kellgren–Lawrence (KL) grade are based on the number of participants (percentage of the whole population). The Mann–Whitney U test was used to compare the mean values of age, BMI, and KOOS scores. The Chi-square test was used to compare the proportions of KL grade between participants with and without EKOA.

*BMI* body mass index, *KOOS* Knee injury and Osteoarthritis Outcome Score, *KL grade* Kellgren–Lawrence grade.

The mean serum MMP-3 concentration was 32.5 ± 10.2 ng/ml in the normal group and 39.0 ± 38.3 ng/ml in the EKOA group (*p* = 0.025), while the other serum biomarkers showed no significant differences (Table 2). The serum MMP-3 concentration had a weak positive correlation with serum IL-6 concentration (r = 0.157, *p* = 0.040) and adiponectin concentration (r = 0.208, *p* = 0.025).

**Table 2:** Laboratory characteristics of participants.

	Total sample (n = 255)	Normal (n = 201)	EKOA (n = 54)	*p* value
MMP-3, ng/mL	34.4 ± 18.4	32.5 ± 10.2	39.0 ± 38.3	0.025
hs-CRP, mg/dL	0.04 ± 0.06	0.04 ± 0.07	0.03 ± 0.03	0.103
IL-6, pg/mL	1.17 ± 2.03	1.05 ± 0.81	1.81 ± 4.61	0.124
Adiponectin, μg/ml	13.0 ± 6.42	12.7 ± 6.43	13.5 ± 6.29	0.925

The serum concentrations of inflammation biomarkers were compared using Mann–Whitney U test. *p* value indicates the significance of difference between participants between normal knees and EKOAs. *MMP-3* matrix metalloproteinases-3, *hs-CRP* high-sensitivity C-reactive protein, *IL-6* interleukin-6.

**Figure 1:**
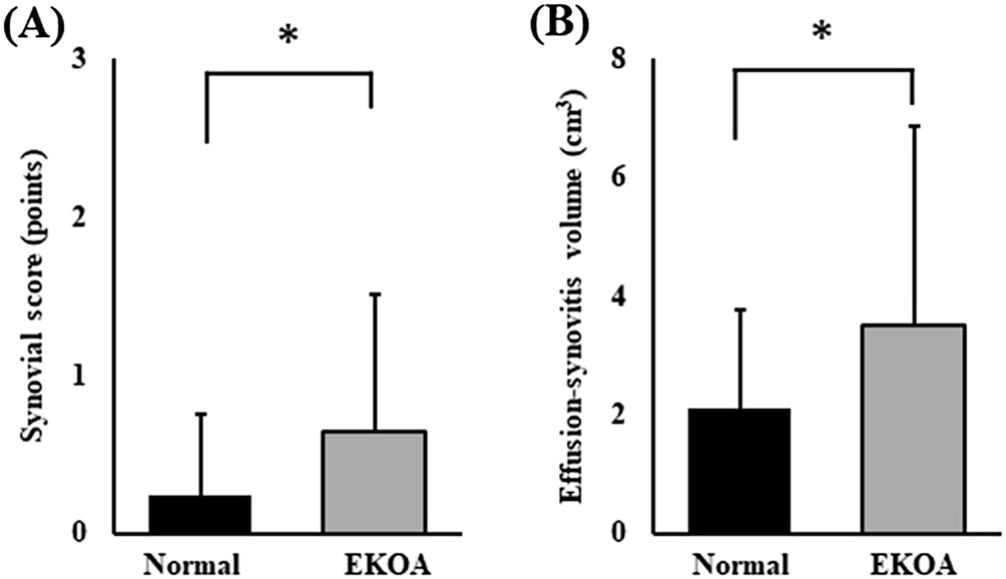
Synovial score and effusion synovitis volume in the normal and EKOA group. Mean values of synovitis score (**a**) and effusion synovitis volume (**b**) between normal and EKOA groups were compared using the Mann–Whitney U test. The error bars represent the standard deviation. A *p*-value below 0.05 was considered significant (*). *EKOA* early knee osteoarthritis.

**Figure 2:**
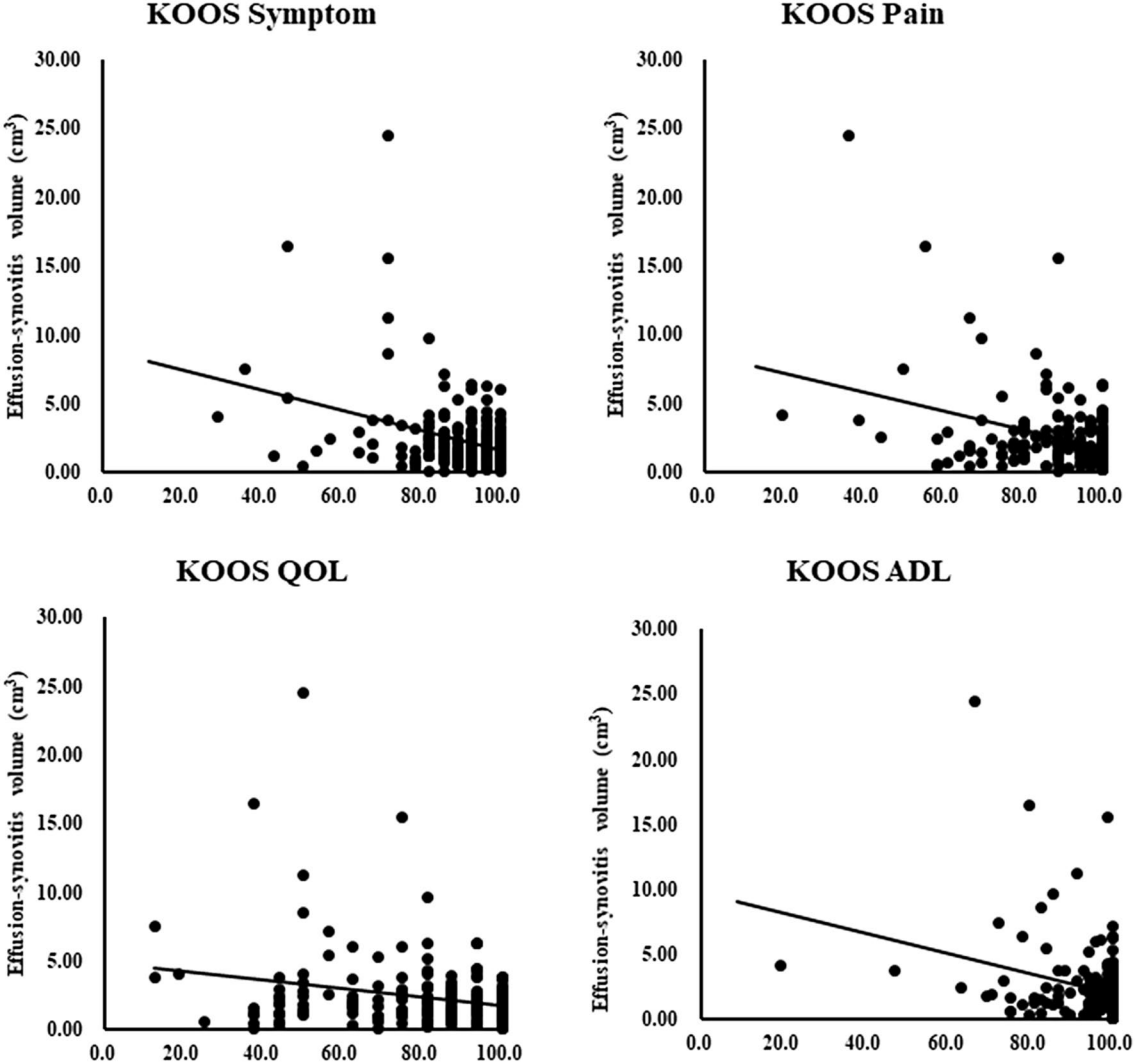
Scatter gram of the effusion-synovitis volume and knee injury and osteoarthritis outcome scale subscales. The x-axis corresponds with each KOOS subscale, and the y-axis corresponds with effusion-synovitis volume (cm^3^) evaluated by magnetic resonance imaging. All subscales of KOOS were negatively correlated with effusion-synovitis volume. *KOOS* knee injury and osteoarthritis outcome scale.

**Figure 3:**
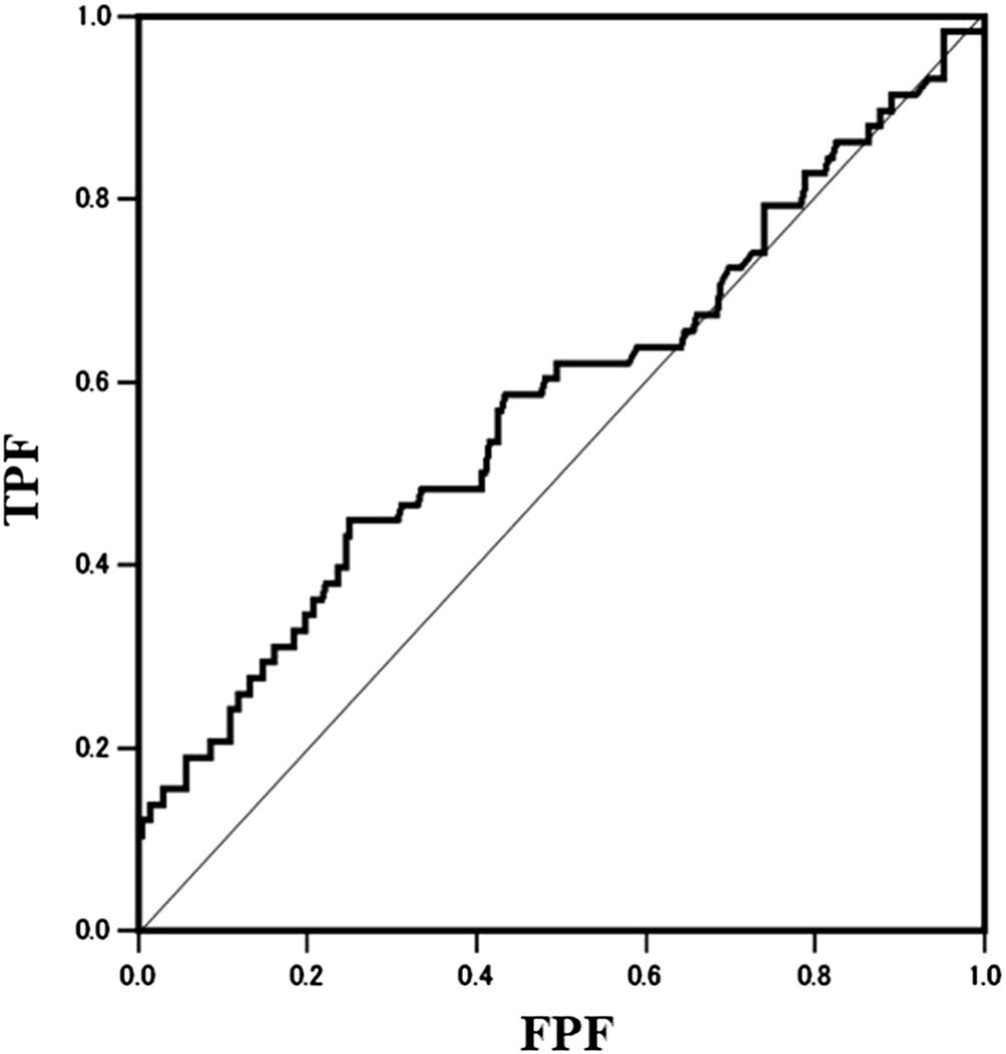
Receiver operating characteristic curves for the effusion-synovitis volume and diagnosing early knee osteoarthritis. *TPF* true-positive fraction, *FPF* false-positive fraction.

The mean synovial score was 0.2 ± 0.4 in the normal group and 0.6 ± 0.9 in the EKOA group; the mean effusion-synovitis volume was 2.0 ± 1.3 cm^3^ in the normal group and 3.5 ± 4.6 cm^3^ in the EKOA group. Both the synovial score and the effusion-synovitis volume in the EKOA group were significantly higher than those in the normal group (*p* = 0.003 and *p* = 0.001, respectively) (Fig. 1). There was a weak positive correlation between serum MMP-3 concentration and effusion-synovitis volume (r = 0.176, *p* = 0.003) (Table 3). The other serum concentrations were not correlated with effusion-synovitis volume (hs-CRP; *p* = 0.181, IL = 6; *p* = 0.203, adiponectin; *p* = 0.437). All KOOS subscales correlated negatively with effusion-synovitis volume (Fig. 2; Table 3). Multivariate regression analysis showed that effusion synovitis volume was associated with serum MMP-3 concentration (*p* < 0.001), but not associated with the other serum inflammation biomarkers (Table 4). According to the ROC analysis, the optimal effusion-synovitis volume cut-off value to detect EKOA (AUC = 0.578; odds ratio: 2.40; *p* = 0.013; Fig. 3) was 2.42 cm^3^. Using this cutoff value, 71 out of 250 patients (28%) demonstrated effusion-synovitis volume of more than 2.42 cm^3^. Among them, 35% of patients (25 of 71) were diagnosed with EKOA; sensitivity was 46% and specificity was 77%.

**Table 3:** Correlations among serum biomarkers, KOOS scores, and effusion synovitis.

	KOOS				Effusion-synovitis area	Synovitis score
	Symptom	Pain	ADL short	QOL		
MMP-3	− 0.008	− 0.008	0.018	− 0.005	0.176**	0.147*
hs-CRP	− 0.144*	− 0.144*	− 0.100	− 0.115	0.020	0.047
IL-6	− 0.094	− 0.094	− 0.090	− 0.136*	0.007	− 0.043
Adiponectin	0.016	0.016	− 0.106	− 0.006	− 0.040	0.030
KOOS symptom		0.630**	0.538**	0.633**	− 0.206**	− 0.276**
KOOS pain			0.761**	0.829**	− 0.155*	− 0.226**
KOOS ADL short				0.732**	− 0.118*	− 0.171**
KOOS QOL					− 0.193**	− 0.265**
Effusion-synovitis area						0.776**
Synovitis score						

Statistical analysis was performed using the Spearman rank correlation coefficient; *p < 0.05; **p < 0.001.

*MMP-3* matrix metalloproteinases-3, *hs-CRP* high-sensitivity C-reactive protein, *IL-6* interleukin-6.

**Table 4:** Univariate and multivariate analyses of the factors associated with effusion-synovitis area detected on MRI.

Parameter	Univariate crude			Multivariate adjusted (stepwise)		
	β	*p*	R^2^	β	*p*	R^2^
Age	− 0.019	0.982	0.064	–	–	–
BMI	0.263	0.020	0.234	0.263	0.020	0.693
MMP-3	0.596	< 0.001	0.320	0.604	< 0.001	0.381
hs-CRP	− 0.010	0.870	0.240	–	–	–
IL-6	− 0.062	0.390	< 0.001	–	–	–
Adiponectin	0.552	0.625	0.281	–	–	–

Crude and adjusted linear regression analyses were performed with effusion-synovitis area as a dependent variable, and serum inflammation biomarker’s concentration, age, and body mass index (BMI) as independent variables. β indicates adjusted correlation coefficient.

MMP-3; matrix metalloproteinases-3, hs-CRP; high-sensitivity C-reactive protein, IL-6; interleukin-6.

## Discussion

This is the first study to report the relationship between serum biomarkers and MRI evaluations in the general Japanese population with EKOA, which was diagnosed based on the new criteria proposed by Luyten et al. Both the mean serum MMP-3 concentrations and the effusion-synovitis volume were higher in the EKOA group than those in the normal group. Multiple regression analysis showed that serum MMP-3 concentration was associated with mild effusion-synovitis detected on MRI. These results suggest that, although not substantial, there is mildly active but definitive synovitis in EKOA. Effusion synovitis may play a role in the etiopathogenic events leading to knee OA and potentially contribute to further progression. MRI could be useful to assess a symptomatic knee joint with no radiographic abnormalities.

Early identification of changes to the knee at an early phase of OA may help prevent the progression to definitive OA. Although many studies had reported the features of early phase of knee OA^1,6,17,18^, there was no clear definition for early phase of knee OA; however, Luyten et al. defined the new criteria^5^. Whilst EKOA is solely diagnosed based on mild knee pain and disabilities, it can be clearly distinguished from both normal and symptomatically abnormal knees. In their cohort study, Sasaki et al. reported that the highest prevalence of EKOA was observed in females aged ≥ 50 years, who had reduced knee function and QOL; the risk factors for EKOA were age, female sex, obesity, and previous knee injury, similar to those of definitive knee OA^6^.

The association of serum biomarkers and EKOA has not been well established. Serum MMP-3 is a diagnostic biomarker for rheumatoid arthritis^19^, and its levels were shown to correlate with synovitis^20^. MMP-3 is produced by synovial membrane cells and chondrocytes in response to increasing mechanical stimulation and exposure to inflammatory cytokines^21^. Although not all investigators agree that there is significant increase in the serum MMP-3 concentrations in patients with OA, Pengas et al. reported that knee synovial MMP-3 concentrations correlated with serum MMP-3 concentrations concluding that serum MMP-3 concentrations could be used as a potential biomarker for knee osteoarthritis and possible disease predictor^22^. Here, the mean serum MMP-3 concentration was higher in the EKOA group than in the normal group.

Previous study demonstrated that serum hs-CRP as well as IL-6 levels increased with the progression of knee OA; the levels of IL-6, but not those of hs-CRP, significantly associated with pain severity^23^. In addition, IL-6 is produced by adipose tissue, which accompanies obesity^24,25^. In the present study, although the mean BMI of participants with EKOA was slightly higher than those with normal knees (*p* = 0.524), serum IL-6 and hs-CRP concentrations showed no significant differences. This might be due to EKOA entailing relatively mild knee joint inflammation. Obesity is a risk factor and onset for knee OA^26,28^. Sowers et al. suggested that the mechanism of knee OA in obese patients might be a simple increase of mechanical burden on the joints^27^. Adiponectin, a 28–30 kDa collagen-like protein, is not only one of the most abundantly secreted adipose tissue proteins but also the only adipokine identified thus far that is negatively correlated with obesity^29^. A recent study found there was a positive correlation between adiponectin concentration and KL grade^26^. On the other hand, a few studies found no statistical association between adiponectin levels and OA^28,30^. Yusuf et al. suggested that the serum adiponectin concentration negatively correlated with the radiographic severity of OA and might play a protective role in the pathogenesis of OA^31^. Our data showed a negative relationship between serum adiponectin concentration and effusion-synovitis volume. These results may support the notion that obesity might be a risk factor for knee joint inflammation.

To date, EKOA’s etiology and pathology remain unclear. Knee OA is a systemic disorder with a multifactorial origin. Previous studies reported MRI features of knee OA without radiographic abnormalities. Harkey et al. reported that the presence of degenerative ligaments, effusion-synovitis, and meniscal pathology served as pre-radiographic structural features, which identified an increased risk of knee OA development over the next 4 years^32^. They also concluded that patients with effusion-synovitis greater than 11.9 cm^3^ were about 3 times more likely to develop knee OA 2 years prior to disease development, with this likelihood increasing to ~ 5.2 times in the year prior to the onset of advanced-stage disease. The CHECK study reported associations between baseline MRI abnormalities and development of radiographic knee OA following 5 years^18^. In the study, cartilage lesions, osteophytes, bone marrow lesions and effusion detected on MRI had significant associations with the progression of definitive knee OA. We showed that effusion-synovitis volume was significantly higher in the EKOA group than that in the normal group, and correlated with all KOOS subscales negatively. From the ROC analysis, the sensitivity was relatively high for detecting EKOA using a cutoff point of 2.42 cm^3^. However, AUC was not high. These results suggested that relatively low active synovitis affects knee pain, and might play one of the important etiological roles of EKOA.

This study has several limitations. First, it was a single-center study, and therefore may be subject to selection bias. For this reason, we instituted strict inclusion and exclusion criteria. Multi-center studies are needed to validate our findings. Second, we assessed knee effusion-synovitis using non-contrast enhanced MRI even though contrast-enhanced MRI (CE-MRI) is considered the gold standard. However, CE-MRI is challenging to apply in large OA studies due to costs, practicality, and rare but possible, side-effects related to the contrast agent^33^. Third, our data showed that serum MMP-3 concentrations significantly correlated with effusion-synovitis volume, but the correlation was relatively weak. Other systemic biomarkers should be investigated. Longitudinal observation of patients with high concentrations of inflammation biomarkers should be performed. Fourth, all participants in this study were women, and the sample size was modest. We performed MRI only on middle-aged women because of the high cost of MRI and because they are the preferred candidates for OA and EKOA. Fifth, KL grade 1 may represent some radiographic abnormalities. A previous study reported that bone attrition, osteophyte formation, and meniscus extrusion were observed in the KL 1 knees^8,17^. However, commonly used radiographic abnormality (knee OA) in a clinical situation was defined by KL grade ≥ 2. In the EKOA group, participants with KL1 knees had a larger synovitis volume than patients with KL0 knees, but without significant difference (*p* = 0.794). Finally, the differences between symptoms of normal ageing and EKOA are controversial^34^. The normal aging process should be taken into consideration while discussing about EKOA. Further longitudinal validation studies are needed. To this end, we are conducting regular follow-ups with the participants of this study to confirm the prognostic power of the new criteria.

Despite these limitations, our study has several important findings that have important clinical relevance concerning intervention for knee pain. We showed the relationship between serum biomarkers and EKOA. The result of MRI and serum biomarkers suggest that synovitis is associated with EKOA.

## Conclusion

The serum MMP-3 concentration, effusion-synovitis volume, and synovial score are valuable metrics for elucidating the characteristics of EKOA. MRI could be used to detect mild synovitis in knees with EKOA. Synovitis might be one of the key factors for intervention in EKOA.

## Materials and methods

### Participants

All participants volunteered for the Iwaki Health Promotion Project, a community-based preventative medicine program that aims to improve the average life expectancy by conducting general health checkups and prophylactic interventions, as previously described^12,13,17,35^. The ethics committee of the our hospital approved the study (reference number: 2017-026), and all of participants gave written informed consent prior to participation.

**Figure 4:**
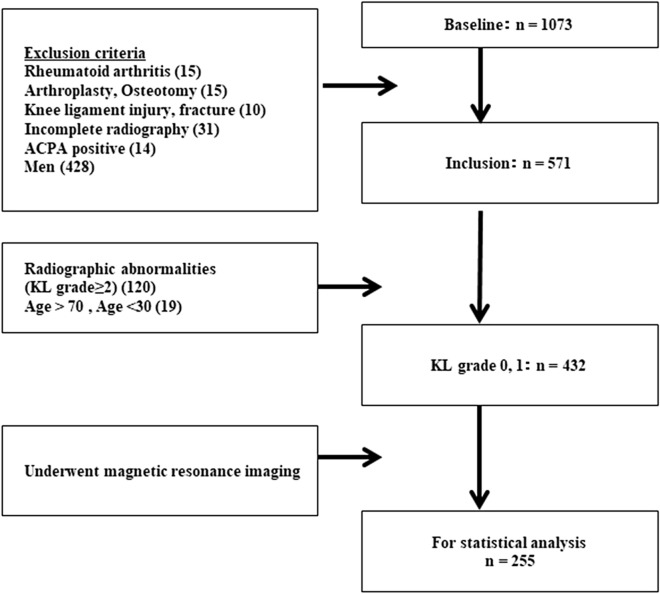
Flow of participants inclusion. Participants enrolled in or excluded from the current study. The values in parentheses indicate the number of excluded participants. *ACPA* anti-cyclic citrullinated peptide antibody, *KL grade* Kellgren–Lawrence grade.

A total of 1,073 volunteers (441 men and 632 women) participated in the project in 2017. Participants answered questionnaires regarding their past and present medical history, lifestyle, occupation, family history, onset of menopause, health-related QOL, and disease-specific information, such as knee symptoms. We focused on female participants aged 30 to 70 years with no radiographic abnormalities because the prevalence of EKOA is higher in middle-aged women^6^. We excluded 15, 31, 10, 15, 14 and 428 participants who had undergone knee arthroplasty, who did not undergo radiographs, with history of knee ligament injury or fracture, diagnosed rheumatoid arthritis, with positive anti-cyclic citrullinated peptide antibody, and who were male participants, respectively. Finally, 255 women who underwent MRI with no radiographic abnormalities (KL glade 0/1) were included in the analysis (Fig. 4).

### Knee injury and osteoarthritis outcome score

Subjective evaluations of knee symptoms were obtained using knee injury and osteoarthritis outcome scores (KOOS), the most frequently used tool that represents patient-based outcome scores^36,37^. KOOS consists of 42 knee-related items, and each item was scored from 0 to 4. Summed scores in 4 subscales (symptom, pain, ADL and QOL) were converted to 100 points as their best condition. Their reliability and internal congruence were high^38^.

### Serum inflammation biomarkers

Blood samples were taken from all participants prior to breakfast in the early morning. Participants had a fasting restriction of 10 h or more prior to having their blood drawn. The serum concentrations of matrix metalloproteinases-3 (MMP-3, ng/mL, CLEIA; LSI Medience Corp., Tokyo, Japan), highly-sensitivity C-reactive protein (hs-CRP, mg/dL, CLEIA; LSI Medience Corp.), interleukin-6 (IL-6, pg/mL, CLEIA; LSI Medience Corp.), and adiponectin (μg/ml, LA; LSI Medience Corp.) were measured for the assessment of inflammation markers.

### Radiographs

Knee radiographic examinations were performed using CXDI-40EG (Canon Inc. Tokyo, Japan). Experienced radiologic technicians and orthopedic surgeons obtained weight-bearing, full extension, and anterior–posterior radiographs of both knees with foot map positioning on the day of the check-up^6,17^. The beam was positioned parallel to the floor with no angle and aimed at the joint space, and the sequencing was set as 60 kV, 50 mA and 80 ms for all participants. A diagnosis of knee OA was defined by KL grade ≥ 2 in the most affected knee. All joints were graded by 2 orthopedic surgeons (DC and ES), and any discrepancy was resolved by mutual consultation. Only the participants showing no radiographic evidence of knee OA were included in this analysis.

### Synovitis evaluated by magnetic resonance imaging

Synovitis on MRI was assessed in their right knee. Participants were positioned supine with their knees in full extension, and scanned with a rapid extremity coil and mobile magnetic resonance unit (1.5 T; ECHELON RX, Hitachi, Tokyo, Japan) within 1 week following other examinations. The scanning sequences were set including sagittal and coronal plane of T2-weighted fat-suppressed fast-spin echo (repetition time 5,000 ms; echo time 80 ms; field of view 16 cm; 288 × 288 matrix; slice thickness of 3 mm with a gap of 1.0 mm between slices). The image database was transferred to an independent computer workstation using the software program OsiriX (Newton Graphic, Inc., Hokkaido, Japan). According to the Whole-Organ MRI Scoring (WORMS) method^39^, synovia were semi-quantitatively graded from 0 to 3 in terms of the estimated maximal distention of the synovial cavity: 0 = normal; 1 = $\le \hspace{0.17em}$33% of maximum potential distention; 2 = 33–66% of maximum potential distention; 3 = $\ge$ 66% of maximum potential distention. Furthermore, effusion-synovitis volume (cm^3^) was quantitatively measured at the suprapatellar fluid equivalent signal on MRI using the software program OsiriX. The ICC (1,1) of effusion synovitis volume was 0.984.

### Classification criteria for early knee osteoarthritis

EKOA was defined according to the classification criteria proposed by Luyten et al.^5^. Classification criteria for EKOA were (A) patient-based questionnaires (KOOS)—2 of the following were required to score “positive” (i.e., ≤ 85%): pain (9 items), symptoms (7 items), activities of daily living (ADL) (short version, 7 items), and knee-related quality of life (QOL) (4 items). (B) Clinical examination—at least one of the following needs to be present: joint line tenderness or crepitus of the knee. (C) Radiographs: KL glade 0/1 at the standing, fixed flexion, and weight bearing positions. Participants were divided into normal knee group (i.e., without EKOA) and EKOA group.

### Statistical analysis

Demographic data among normal and EKOA groups are expressed as mean ± standard deviation. The Chi-square test was performed for categorical variables and Mann–Whitney U test was performed for continuous variables to compare demographic data between groups, due to some demographic parameters not being normally distributed according to the Shapiro–Wilk test. The serum concentrations of inflammation biomarkers were compared using the Mann–Whitney U test. The correlations of serum concentrations of each of the inflammation biomarkers were analyzed using the Spearman rank correlation.

The synovial scores and effusion-synovitis volumes were compared using the Mann–Whitney U test; the relationship between serum concentrations of inflammation biomarker and effusion-synovial volume were analyzed using the Spearman rank correlation; to examine associations between knee symptoms and synovitis, Spearman rank correlation was performed between each KOOS score and effusion synovitis volume; to examine the associations between effusion-synovitis volume and inflammation biomarkers, multiple linear regression analyses were performed with effusion-synovitis volume as dependent variables, and serum inflammation biomarkers, age, and BMI as independent variables; finally, to estimate the predictive cutoff level of effusion-synovitis for diagnosing the EKOA, receiver operating characteristic (ROC) analysis was performed. The values of effusion-synovitis volume were used as a single variable in this analysis. A false positive fraction was plotted against the 1 true positive fraction, and the cutoff point was defined as the point of the maximum slope, i.e., the nearest point to true positive. The volume under the curve (AUC) was calculated to evaluate the validity of the ROC analysis. Data input and analysis were performed using SPSS version 25.0J (SPSS Inc., Chicago, IL, USA). A *p* value < 0.05 was considered statistically significant.

### Ethical approval

All participants provided written informed consent, and the study was done in agreement with the 1964 Helsinki declaration and its later amendments or comparable ethical standards and conducted with the approval of the ethics committee of Hirosaki University Graduate School of Medicine.

### Consent to participate

Informed consent was obtained from all individual participants included in the study.

### Consent for publication

Not applicable.
